# Retinol and Pro-Vitamin A Carotenoid Nutritional Status during Pregnancy Is Associated with Newborn Hearing Screen Results

**DOI:** 10.3390/nu15040800

**Published:** 2023-02-04

**Authors:** Rebecca Slotkowski, Matthew Van Ormer, Anum Akbar, Olivia Paetz, Taija Hahka, Maranda Thompson, Alyssa Freeman, Alexandra Hergenrader, Sarah Sweeney, Zeljka Korade, Thiago Genaro-Mattos, Corrine Hanson, Ann Anderson-Berry, Melissa Thoene

**Affiliations:** 1 Department of Pediatrics, University of Nebraska Medical Center, Omaha, NE 68198, USA; 2 Munroe Meyer Institute, University of Nebraska Medical Center, Omaha, NE 68198, USA; 3 Medical Nutrition Education Program, College of Allied Health Professions, University of Nebraska Medical Center, Omaha, NE 68198, USA

**Keywords:** carotenoids, retinol, β-carotene, pregnancy, neonatal, hearing, newborn hearing screen

## Abstract

The prenatal period is critical for auditory development; thus, prenatal influences on auditory development may significantly impact long-term hearing ability. While previous studies identified a protective effect of carotenoids on adult hearing, the impact of these nutrients on hearing outcomes in neonates is not well understood. The purpose of this study is to investigate the relationship between maternal and umbilical cord plasma retinol and carotenoid concentrations and abnormal newborn hearing screen (NHS) results. Mother–infant dyads (*n* = 546) were enrolled at delivery. Plasma samples were analyzed using HPLC and LC–MS/MS. NHS results were obtained from medical records. Statistical analysis utilized Mann–Whitney U tests and logistic regression models, with *p* ≤ 0.05 considered statistically significant. Abnormal NHS results were observed in 8.5% of infants. Higher median cord retinol (187.4 vs. 162.2 μg/L, *p* = 0.01), maternal *trans*-β-carotene (206.1 vs. 149.4 μg/L, *p* = 0.02), maternal *cis*-β-carotene (15.9 vs. 11.2 μg/L, *p* = 0.02), and cord *trans*-β-carotene (15.5 vs. 8.0 μg/L, *p* = 0.04) were associated with abnormal NHS. Significant associations between natural log-transformed retinol and β-carotene concentrations and abnormal NHS results remained after adjustment for smoking status, maternal age, and corrected gestational age. Further studies should investigate if congenital metabolic deficiencies, pesticide contamination of carotenoid-rich foods, maternal hypothyroidism, or other variables mediate this relationship.

## 1. Introduction

Pro-vitamin A carotenoids, including α-carotene, β-carotene, and β-cryptoxanthin, are anti-inflammatory nutrients found in vegetables, fruits, and fish which can be converted into vitamin A in the body [1,2,3]. Vitamin A plays an important role in pregnancy and fetal development by regulating normal organogenesis, tissue differentiation, and development of the immune system and the inner ear [4]. Furthermore, numerous studies have shown that the antioxidant and anti-inflammatory properties of carotenoids can ameliorate pregnancy-associated morbidities such as pre-eclampsia, intrauterine growth restriction, gestational diabetes, and pregnancy induced hypertension [5,6,7,8]. However, vitamin A deficiency is still prevalent and is considered a worldwide public health problem, affecting an estimated 19 million pregnancies every year [9]. This deficiency is associated with long-term health consequences for the infant, including hearing loss [10].

Vitamin A is indispensable in inner ear development. During in utero development, essential enzymes in the inner ear convert vitamin A (retinol) into a biologically active form, retinoic acid. Previous studies reported that retinoic acid regulates fibroblast growth factors (FGF) such as FGF3 and FGF10, which may in turn modulate several downstream target molecules that are necessary for normal inner ear development [11,12]. Surface ectoderm gives rise to the otic placode during the first trimester of gestation, which in turn forms the mature inner ear. Studies have shown that vitamin A induces development of the otic placode, which further corroborates the role of vitamin A in inner ear development. In fact, experiments in rats, zebrafish, and chicks demonstrate that deficiency of vitamin A leads to aberrant otic placode development [12,13].

Developmental defects of the inner ear, which is composed of the cochlear and vestibular systems, are a major cause of congenital sensorineural hearing loss [14,15] and approximately one-third of children with congenital sensorineural hearing loss have significant hearing impairment [16]. The American Academy of Pediatrics recommends that a newborn hearing screen (NHS) should be done within the first month of life to detect congenital sensorineural hearing loss [17]. There are several known causes of congenital hearing loss including genetic factors and congenital infections (such as congenital cytomegalovirus, toxoplasmosis, and rubella infection). Early detection of and intervention for congenital sensorineural hearing loss is important, as previous studies have shown that children diagnosed with congenital sensorineural hearing loss earlier have better language and socioemotional outcomes, likely resulting from early interventions [18]. The protocols for NHS vary in different countries; in the United States, NHS may include otoacoustic emissions (OAE) or automated auditory brainstem response (AABR) tests [19]. OAE is helpful in assessing the inner ear (cochlear function) whereas automated AABR assesses the auditory pathway in addition to assessing the function of the external and inner ear [20,21]. This comprehensive screening of newborns reduces the chance of missing auditory neuropathy spectrum disorders [22]. However, although previous studies have demonstrated that vitamin A is required for normal ear development, there is a gap in our knowledge regarding how maternal and infant nutritional status of pro-vitamin A carotenoids are associated with the results of these newborn hearing screens.

To address this gap, we conducted a study to assess the relationship between NHS results and retinol and pro-vitamin A carotenoid maternal intake, maternal plasma levels, and umbilical cord plasma levels. As pro-vitamin A carotenoids can be converted into vitamin A and vitamin A plays a key role in inner ear development, we hypothesized that higher concentrations of retinol, α-carotene, β-carotene, and β-cryptoxanthin in maternal and umbilical cord plasma would correlate with better NHS outcomes.

## 2. Materials and Methods

### 2.1. Participant Enrollment

Ethical approval for this study was obtained from the University of Nebraska Medical Center Institutional Review Board (#112-15-EP). Eligible mothers were enrolled at the time of delivery (*n* = 546). Mothers provided written consent for both themselves and their infant(s) prior to participation. Inclusion criteria included women ≥ 19 years old admitted to the Labor and Delivery Unit at Nebraska Medicine during the study period (June 2015 to August 2021) who delivered at least one live-born infant. Exlcusion criteria included gastrointestinal, liver, or kidney diseases which would affect normal nutrient metabolism, inborn errors of metabolism, congenital abnormalities in the infant, and infants who were deemed wards of the state.

### 2.2. Biological Sample Collection

Maternal blood samples were collected in K2 EDTA tubes during routine labs upon admission for delivery. Umbilical cord blood samples were collected in K2 EDTA tubes during routine labs at the time of delivery. Whole blood samples were protected from heat and light to preserve nutrient integrity. Samples were seperated into plasma and frozen at −80 °C within a maximum of 12 h after collection. Blood samples were available for 404 maternal–infant dyads.

### 2.3. Carotenoid Laboratory Analysis

Plasma samples (*n* = 404) were analyzed for retinol and pro-vitamin A carotenoids (α-carotene, β-carotene, and β-cryptoxanthin). The first 328 dyad plasma samples were analyzed at the Biomarker Research Institute at the Harvard T.H. Chan School of Public Health via HPLC as previously described by Thoene et al. [23]. The remaining 76 dyad plasma samples were analyzed at the University of Nebraska Medical Center via LC–MS/MS as previously described by McConnell et al. [24]. Quality control at both labs was achieved using NIST standards.

### 2.4. Dietary Intake and Socioeconomic Questionnaires

The Harvard Food Frequency Questionnaire (FFQ) [25] was administered to maternal participants at the time of delivery by trained study personnel. The FFQ was completed for 472 participants. De-identified FFQs were analyzed by the Harvard T.H. Chan School of Public Health to quantify average daily nutrient intake from foods and supplements over the previous year. The FFQ is validated for use in pregnant women [26]. Women with estimated daily caloric intakes outside a probable range (800–8000 Cal/day) were excluded from analysis [27].

A socioeconomic status questionnaire collected information on annual household income and household size prior to delivery of the infant (*n* = 363). The income-to-poverty ratio was calculated by dividing the reported annual household income by the federal poverty level for the reported household size [28].

### 2.5. NHS Results and Clinical Data Collection

NHS were conducted using automated auditory brainstem response (AABR; NATUS ALGO^®^ 5 Newborn Hearing Screener) prior to infant discharge from the Newborn Nursery or Neonatal Intensive Care Unit. To minimize the rate of falsely abnormal AABR results [29], NHS were conducted per standard of care guidelines. Initial NHS results were collected from the infant electronic medical record (EMR). Additional variables collected from the infant EMR included corrected gestational age at birth (CGA), sex, NICU admission, birthweight, days of antibiotic therapy, and days of oxygen therapy. Maternal age, race/ethnicity, and smoking status were collected from the maternal EMR.

### 2.6. Statistical Analysis

Descriptive statistics were calculated, including medians and interquartile ranges for continuous variables and frequencies and percentages for categorical variables. Retinol intake was categorized as insufficient (≤770 μg RAE/day), adequate (771–2999 μg RAE/day), or excessive (≥3000 μg RAE/day) according to Institute of Medicine guidelines [30] and retinol plasma concentrations were categorized as deficient (≤200.5 μg/L), insufficient (200.5–300.8 μg/L), or adequate (>300.8 μg/L) according to the World Health Organization guidelines [9]. Chi-square tests were used to identify differences in categorical retinol nutritional status between normal vs. abnormal NHS groups. Mann–Whitney U tests were used to identify differences in median retinol and pro-vitamin A carotenoids intake/plasma concentrations between normal vs. abnormal NHS groups. Logistic regression models were used to evaluate the association between natural-log transformed plasma nutrient concentrations and NHS results, with adjustment for CGA, maternal age, and smoking status (Model 1) or CGA, maternal age, smoking status, and income-to-poverty ratio (Model 2). Odds ratios with 95% confidence intervals (95% CI) are reported for a 1-unit increase in natural log-transformed nutrient concentrations (OR_ln_ = e^B^; 95% CI = e^B ± 1.96SE^) and for a 10% increase in nutrient plasma concentrations (not natural-log transformed; OR_10%_ = 1.1^B^; 95% CI = 1.1^B ± 1.96SE^) [31]. A 1-unit increase in natural log-transformed nutrient concentration is equivalent to a 172% increase in nutrient plasma concentration (not natural-log transformed). Subjects with non-detectable plasma concentrations or missing maternal intake information were excluded from the corresponding analysis. A *p*-value ≤ 0.05 was considered statistically significant. For twin pregnancies, mothers and twin A were included in the analysis; twin B was excluded.

## 3. Results

### 3.1. Demographic Characteristics

A total of 546 mother–infant dyads participated in this study, including 47 (8.6%) infants with abnormal NHS results and 499 (91.4%) with normal NHS results. The demographic characteristics of the sample population by NHS result are shown in Table 1. Data on CGA and maternal age was available for all participants. Income-to-poverty ratio and energy intake was available for 363 (330 normal, 33 abnormal NHS) and 472 (433 normal, 39 abnormal NHS) maternal–infant dyads, respectively. Although very low birth weight (≤1500 g), administration of antibiotics, and hypoxia (measured as days of oxygen therapy) have previously been associated with hearing loss in infants [32], these factors were rarely present in our population of infants with abnormal NHS.

### 3.2. Retinol Nutritional Status and NHS Results

Maternal retinol intake over the past year was categorized into insufficient, adequate, or excessive using the Institute of Medicine guidelines [30]. Maternal and infant retinol plasma concentrations were categorized into deficient, insufficient, or adequate using WHO guidelines [9]. There were no significant associations between either retinol intake groups or retinol plasma status categories and NHS result groups (Table 2).

However, median cord retinol plasma concentrations were significantly higher among infants with abnormal NHS compared to those with normal NHS (*p* = 0.01; Table 3). After adjustment for CGA, maternal age, and smoking status, natural log-transformed cord plasma concentrations of retinol remained significantly associated with abnormal NHS (Table 4; Model 1). A 10% increase in cord retinol plasma concentrations was associated with 1.15 (95% CI 1.05, 1.27; *p* = 0.01) times higher odds of abnormal NHS (Table 4; Model 1). Poverty-to-income ratio was available on a subset of maternal–infant dyads. After additional adjustment for income-to-poverty ratio, a 10% increase in cord retinol plasma concentration was associated with 1.20 (95% CI 1.05, 1.36; *p* = 0.01) times higher odds of abnormal NHS results (Table 4; Model 2). There were no significant differences in maternal plasma retinol concentrations or maternal retinol intake between groups.

### 3.3. β-Carotene Intake, Maternal Plasma, and Cord Plasma Concentrations

Median maternal plasma concentrations of trans-β-carotene (*p* = 0.02) and cis-β-carotene (*p* = 0.02) were significantly higher in mothers of infants with abnormal NHS results compared to mothers of infants with normal NHS results (Table 5). Cord plasma concentrations of trans-β-carotene (*p* = 0.02) and total β-carotene (*p* = 0.04) were significantly higher in infants with abnormal NHS results. Maternal β-carotene intake during pregnancy was not significantly different between mothers of infants with normal NHS versus abnormal NHS results.

After adjustment for CGA, maternal age, and smoking status, natural log-transformed maternal trans-β-carotene (95% CI 1.00, 1.11; *p* = 0.04), maternal cis-β-carotene (95% CI 1.00, 1.11; *p* = 0.04), cord trans-β-carotene (95% CI 1.01, 1.12; *p* = 0.03), and cord total β-carotene (95% CI 1.02, 1.10; *p* = 0.003) remained significantly associated with abnormal NHS (Table 6; Model 1). After additional adjustment for poverty-to-income ratio, maternal trans-β-carotene (95% CI 1.02, 1.18; *p* = 0.02), maternal cis-β-carotene (95% CI 1.02, 1.18; *p* = 0.02), and cord total β-carotene (95% CI 1.00, 1.10; *p* = 0.05) remained significantly associated with abnormal NHS results (Table 6; Model 2).

### 3.4. Intake, Maternal Plasma, and Cord Plasma Concentration of α-carotene and β-cryptoxanthin

No significant differences in α-carotene or β-cryptoxanthin intake, maternal plasma concentrations, or infant plasma concentrations were observed between NHS groups (Table 7).

## 4. Discussion

Contrary to our original hypothesis, we detected that higher infant plasma concentrations of retinol, as well as higher maternal and infant plasma concentrations of β-carotene, were associated with increased odds of abnormal NHS. Other studies investigating the association between retinol and carotenoid nutritional status and hearing loss have focused on intake in adult populations, rather than plasma concentrations in infants. Although not statistically significant, median maternal intake of β-carotene was also higher for dyads in this study with an abnormal NHS result (5570.9 vs. 4881.8 μg/day, *p* = 0.15). These findings are in stark contrast to studies in adult populations that have demonstrated an opposite effect, with increased β-carotene intake being associated with a decreased likelihood of acquired hearing loss [33,34,35]. Differences in population age and mechanism of hearing loss (congenital vs. acquired) between our study cohort and previous studies in adult populations could explain our divergent findings.

Studies examining the relationship between acquired hearing loss and retinol in children, rather than adults, have reported mixed results. One randomized placebo-controlled trial by Schmitz et al. reported that supplementation with vitamin A during preschool was associated with a decreased risk of hearing impairment during adolescence [36]. In contrast, a randomized placebo-controlled trial by Ambalavanan et al. reported that there was no difference in the risk of infant hearing impairment at 18 to 22 months adjusted age between extremely low birth weight infants who receive vitamin A supplementation compared to those who received placebo [37]. Similar to Ambalavanan et al., we observed no association between maternal retinol intake and odds of infant hearing impairment as measured by NHS results. However, infant plasma concentrations of both retinol and β-carotene were associated with increased odds of abnormal NHS results in our cohort.

There are several potential explanations for the observed association between increased retinol and β-carotene plasma concentrations and increased odds of abnormal NHS. Excessive maternal vitamin A intake during gestation is associated with abnormal fetal inner ear development in both humans and animal models [12,38]. The Institute of Medicine Food and Nutrition Board recommends 770 μg RAE/day of vitamin A, with a tolerable upper limit of 3000 μg RAE/day for preformed vitamin A [30]; however, intake of up to 9000 μg RAE/day of preformed vitamin A, almost twelve times the recommended daily dose and three times the tolerable upper limit, has been shown to be safe during pregnancy [39]. Additionally, although β-carotene can be metabolized into vitamin A, excessive intake of β-carotene is not thought to result in vitamin A toxicity during pregnancy [39]. In this study, median maternal intake of retinol for the mothers of infants with abnormal NHS results over the past year was 1,755.9 μg RAE/day, well within recommended limits [30]. Among mothers of infants with abnormal NHS results, only three (7.7%) consumed less than 770 μg RAE/day and only two (5.1%) consumed more than 3000 μg RAE/day. These intakes are comparable to mothers of infants with normal NHS results, where 9.9% consumed less than 770 μg RAE/day and 6.2% consumed more than 3000 μg RAE/day. Likewise, although most infants in this study had insufficient or deficient retinol plasma concentrations of retinol, the median retinol plasma concentration was significantly higher among infants with an abnormal NHS.

Alternatively, it is possible that impaired vitamin A metabolism or transport during inner ear development may be associated with both congenital hearing loss and increased plasma concentrations of retinol and β-carotene. During normal inner ear development, β-carotene is converted to retinol [1,2,3], and retinol is converted to the biologically active retinoic acid [11,12]. Retinoic acid is then utilized in signaling cascades to promote inner ear development [11,12]. Impaired metabolism of retinol into retinoic acid could result in accumulation of plasma retinol. A high concentration of retinol could, in turn, impair metabolism of β-carotene into retinol and result in accumulation of plasma β-carotene. Similarly, an imbalance in retinol-binding protein could result in impaired transport of retinol to target tissues, leading to accumulation of retinol, and ultimately β-carotene, in plasma. However, vitamin A plays an important role in organogenesis, tissue differentiation, and immune system development, in addition to inner ear development [4]. It is unlikely that impaired metabolism of vitamin A would result in congenital hearing loss without significantly affecting the development of other organ systems. In this study, 87% of infants with an abnormal NHS result were otherwise healthy infants who were not admitted to the NICU at delivery, though long-term health of these infants has not been evaluated.

Another potential explanation for the observed association between retinol and β-carotene and abnormal NHS is that infants with higher retinol and β-carotene plasma concentrations may have been exposed to higher levels of ototoxic environmental contaminants in vitamin A and carotenoid-rich foods, such as pesticides applied to fruits and vegetables. We observed a trend towards higher median maternal β-carotene intake in mothers of infants with an abnormal NHS result, which may support this theory, although the relationship was not significant and there were similarly no significant differences in maternal intake of other evaluated carotenoids. Multiple studies have reported associations between pesticide or other environmental contaminant exposure and hearing loss [40,41,42,43,44,45]. In a rat model, prenatal exposure to the environmental contaminants 2,3,7,8-tetrachorodibenzo-p-dioxin (TCDD) [40] or polychlorinated biphenyls (PCB) [41,42] were associated with hearing deficits. In humans, higher prenatal exposure to multiple organochlorine pesticides, as measured in cord plasma concentrations, were associated with significantly worse cochlear function, as measured by distortion product otoacoustic emission (DPOAE) at 45 months of age [43].

Alternative rationales may exist for these findings, including variables unknown or not evaluated. For example, metabolic and endocrine disorders such as hypothyroidism [46,47,48,49], diabetes [50,51], and polycystic ovarian syndrome (PCOS) [52,53] have been associated with both hearing loss and alterations in vitamin A nutritional status. Subclinical hypothyroidism is particularly common during pregnancy, affecting an estimated 15.5% of pregnant women in the United States [54]. Interestingly, hypothyroidism is associated with increased concentrations of β-carotene [46,47]. Maternal hypothyroidism and congenital hypothyroidism are also linked to neurosensory hearing loss [48,49], although less is known about how subclinical hypothyroidism may affect infant hearing. One study by Radetti et al. failed to detected any significant association between maternal subclinical hypothyroidism and infant hearing outcomes [55], while a study by G et al. observed that infants born to mothers with subclinical hypothyroidism had minor alterations in hearing which self-corrected within 6–8 months [56]. As the American Thyroid Association does not currently recommend asymptomatic screening for subclinical hypothyroidism during pregnancy [57], thyroid function was not evaluated in this study. However, it is possible that maternal–infant dyads with subclinical hypothyroidism may jointly exhibit higher β-carotene concentrations and newborn hearing screen fails, though these results would not indicate a causal relationship between nutritional status of β-carotene and infant NHS results. Future research is therefore needed to evaluate the relationship between these variables.

### Limitations

This study was conducted at a single academic medical center in the Midwest United States (University of Nebraska Medical Center/Nebraska Medicine) with a majority non-Hispanic White cohort of maternal–infant dyads, which may limit generalizability of our results. We were unable to collect information on some factors that have been previously associated with hearing loss in infants, such as hyperbilirubinemia, craniofacial abnormalities, administration of loop diuretics, or various environmental exposures (e.g., viral infections) [32]. However, our analysis did account for several other variables potentially associated with neonatal hearing loss, including gestational age of the infant, maternal age, maternal smoking status, and income-to-poverty ratio. Additionally, this analysis focused on associations between first NHS result and nutritional status at time of delivery. Neonates may have abnormal NHS results which resolve on repeat testing and nutritional status at time of delivery may differ from nutritional status during the critical period of inner ear development in the first trimester of pregnancy. Future studies should assess associations between diagnosed congenital hearing loss and retinol and β-carotene nutritional status across multiple timepoints in pregnancy.

## 5. Conclusions

The observed relationship between higher retinol and β-carotene infant plasma concentrations and increased odds of abnormal NHS was unexpected. While other studies suggest both deficient and excessive levels of vitamin A can impact inner ear development, median retinol plasma levels in our study were within normal limits. One possible explanation for our observed results is that higher neonatal retinol and β-carotene plasma concentrations may be indicative of impaired retinol metabolism or transport to the inner ear. Alternatively, exposure to ototoxic environmental contaminants, maternal metabolic disorders, maternal endocrine disorders, perinatal infections, or other variables not assessed in this study could be associated with both increased retinol and β-carotene plasma concentrations and higher odds of abnormal NHS results. Future research should evaluate the association between plasma retinol and β-carotene concentrations and diagnosed congenital hearing loss in a larger cohort of maternal–infant dyads to further investigate this relationship.

## Figures and Tables

**Table 1:** Participant characteristics by NHS result.

	Total Population	Normal NHS	Abnormal NHS
Median CGA (Weeks; IQR)	39.3 (38.0–40.3)	39.3 (37.7–40.1)	39.4 (38.6–40.6)
Median maternal age (Years; IQR)	29.0 (25.0–33.0)	29.0 (25.0–33.0)	29.0 (23.0–32.0)
Median income-to-poverty ratio (IQR)	2.7 (1.2–3.6)	2.7 (1.3–3.6)	1.8 (0.9–3.6)
Median maternal energy intake (kcals/day; IQR)	2058 (1641–2644)	2061 (1635–2664)	1939 (1715–2518)
Race/Ethnicity (*n*; %)			
Non-Hispanic White	363 (66.5)	333 (66.7)	30 (63.8)
Non-Hispanic Black	82 (15.0)	71 (14.2)	11 (23.4)
Non-Hispanic Asian/Pacific Islander	12 (2.2)	11 (2.2)	1 (2.1)
Hispanic	40 (7.3)	36 (7.2)	4 (8.5)
Other/unknown	49 (9.0)	48 (9.6)	1 (2.1)
Smoking Status (*n*; %)			
Never smoker	418 (76.6)	382 (76.6)	36 (76.6)
Former/current smoker	128 (23.4)	117 (23.4)	11 (23.4)
NICU Admission (*n*; %)			
Newborn Nursery	407 (74.5)	366 (73.3)	41 (87.2)
NICU	139 (25.5)	133 (26.7)	6 (12.8)
Infant Sex (*n*; %)			
Female	260 (47.6)	239 (47.9)	21 (44.7)
Male	286 (52.4)	260 (52.1)	26 (55.3)
Birth Weight (*n*; %)			
>1500 g	533 (97.6)	487 (97.6)	46 (97.9)
≤1500 g	13 (2.4)	12 (2.4)	1 (2.1)
Antibiotic Days (*n*; %)			
No antibiotics	500 (91.6)	456 (91.4)	44 (93.6)
≥1 day of antibiotics	46 (8.4)	43 (8.6)	3 (6.4)
Oxygen Therapy Days (*n*; %)			
No oxygen	483 (88.5)	439 (88.0)	44 (93.6)
≥1 day of oxygen	63 (11.5)	60 (12.0)	3 (6.4)

**Table 2:** Categorical retinol intake, maternal plasma, and cord plasma by NHS result.

	Normal NHS Results	Abnormal NHS Results	
	*n* (%)	*n* (%)	*p*-Value ^1^
Total Retinol Intake			0.86
Insufficient (<770 μg RAE/day)	43 (9.9)	3 (7.7)	
Adequate (770–3000 μg RAE/day)	363 (83.8)	34 (87.2)	
Excessive (>3000 μg RAE/day)	27 (6.2)	2 (5.1)	
Maternal Plasma Retinol			0.10
Deficient (<200.5 μg/L)	35 (9.6)	4 (12.5)	
Insufficient (200.5–300.8 μg/L)	149 (41.0)	7 (21.9)	
Adequate (>300.8 μg/L)	179 (49.3)	21 (65.6)	
Cord Plasma Retinol			0.12
Deficient (<200.5 μg/L)	247 (71.0)	15 (53.6)	
Insufficient (200.5–300.8 μg/L)	84 (24.1)	10 (35.7)	
Adequate (>300.8 μg/L)	17 (4.9)	3 (10.7)	

^1^ Chi-square test comparing retinol intake between normal and abnormal NHS.

**Table 3:** Continuous retinol intake, maternal plasma, and cord plasma by NHS result.

	Normal NHS Results		Abnormal NHS Results		
	*n*	Median (IQR)	*n*	Median (IQR)	*p*-Value ^1^
Total retinol intake (μg RAE/day)	433	1746.9 (1113.9–2189.5)	39	1755.9 (1110.9–2254.6)	0.89
Retinol intake without supplements (μg RAE/day)	433	896.4 (650.9–1180.2)	39	877.3 (658.5–1373.6)	0.63
Retinol supplements (μg RAE/day)	433	900.0 (171.4–1200.0)	39	1200.0 (171.4–1200.0)	0.67
Maternal plasma retinol (μg/L)	363	300.0 (247.3–366.1)	32	344.5 (269.5–427.7)	0.08
Cord plasma retinol (μg/L)	348	162.1 (130.3–209.0)	28	187.4 (159.6–261.6)	0.01

^1^ Mann–Whitney U test comparing retinol nutritional status between normal and abnormal NHS.

**Table 4:** Multivariate binary logistic regression predicting abnormal NHS result using natural log-transformed plasma retinol concentrations.

Model 1 ^1^						
	*n*	B	SE	OR_ln_ (95% CI) ^3^	OR_10%_ (95% CI) ^4^	*p*-Value
Ln-maternal retinol	395	1.08	0.58	2.95 (0.94–9.25)	1.11 (0.99–1.24)	0.06
Ln-cord retinol ^1^	376	1.51	0.52	4.52 (1.64–12.51)	1.15 (1.05–1.27)	0.01
**Model 2 ^2^**						
	***n***	**B**	**SE**	**OR_ln_ (95% CI) ^3^**	**OR_10%_ (95% CI) ^4^**	***p*-Value**
Ln-maternal retinol	238	0.57	0.79	1.77 (0.38–8.24)	1.06 (0.91–1.22)	0.47
Ln-cord retinol ^1^	227	1.87	0.69	6.50 (1.68–25.22)	1.20 (1.05–1.36)	0.01

^1^ Model 1 is adjusted for CGA, maternal age, and smoking status. ^2^ Model 2 is adjusted for CGA, maternal age, smoking status, and income-to-poverty ratio. ^3^ Odds ratio for abnormal NHS results assuming 1-unit increase in ln(plasma retinol). ^4^ Odds ratio for abnormal NHS results assuming 10% increase in plasma retinol.

**Table 5:** β-carotene intake, maternal plasma, and cord plasma concentrations by NHS result.

	Normal NHS Result		Abnormal NHS Result		
	*n*	Median (IQR)	*n*	Median (IQR)	*p*-Value ^1^
Maternal Intake (μg/day)					
Total β-carotene	433	4963.6 (3176.6–7398.9)	39	5570.9 (3021.2–8755.5)	0.28
β-carotene without supplements	433	3952.7 (2276.1–6267.5)	39	4619.9 (2123.5–7759.4)	0.45
β-carotene supplements	433	342.9 (85.7–2400.0)	39	660.0 (21.4–2400.0)	0.31
Maternal Plasma (μg/L)					
Trans-β-carotene	301	148.7 (67.9–252.1)	21	206.1 (140.7–523.8)	0.02
Cis-β-carotene	296	11.2 (5.8–20.6)	21	15.9 (11.0–43.0)	0.02
Total β-carotene	363	147.1 (70.4–277.6)	32	195.6 (81.5–385.7)	0.14
Cord Plasma (μg/L)					
Trans-β-carotene	279	8.1 (4.8–15.7)	17	15.6 (6.9–20.7)	0.02
Cis-β-carotene	208	2.0 (1.2–3.4)	11	3.2 (1.6–4.9)	0.06
Total β-carotene	339	9.4 (5.4–17.8)	26	15.9 (6.4–36.1)	0.04

^1^ Mann–Whitney U test comparing β-carotene nutritional status between normal and abnormal NHS.

**Table 6:** Multivariate binary logistic regression predicting abnormal NHS result using natural log-transformed plasma β-carotene concentrations.

Model 1 ^1^						
	*n*	B	SE	OR_ln_ (95% CI) ^3^	OR_10%_ (95% CI) ^4^	*p*-Value
Maternal Plasma						
Ln-trans-β-carotene	322	0.59	0.28	1.80 (1.04–3.13)	1.06 (1.00–1.11)	0.04
Ln-cis-β-carotene	317	0.59	0.28	1.80 (1.04–3.13)	1.06 (1.00–1.11)	0.04
Ln-total β-carotene	395	0.23	0.20	1.26 (0.86–1.85)	1.02 (0.98–1.06)	0.24
Cord Plasma						
Ln-trans-β-carotene	296	0.61	0.28	1.84 (1.06–3.18)	1.06 (1.01–1.12)	0.03
Ln-cis-β-carotene	219	0.72	0.38	2.05 (0.97–4.35)	1.07 (1.00–1.15)	0.06
Ln-total β-carotene	365	0.61	0.20	1.83 (1.24–2.71)	1.06 (1.02–1.10)	0.003
**Model 2 ^2^**						
	***n***	**B**	**SE**	**OR_ln_ (95% CI) ^3^**	**OR_10%_ (95% CI) ^4^**	***p*** **-Value**
Maternal Plasma						
Ln-trans-β-carotene	188	0.97	0.39	2.63 (1.22–5.67)	1.10 (1.02–1.18)	0.02
Ln-cis-β-carotene	185	0.94	0.39	2.55 (1.18–5.50)	1.09 (1.02- 1.18)	0.02
Ln-total β-carotene	238	0.03	0.23	1.03 (0.65–1.62)	1.00 (0.96–1.05)	0.92
Cord Plasma						
Ln-trans-β-carotene	176	0.59	0.34	1.81 (0.93–3.54)	1.06 (0.99–1.13)	0.08
Ln-cis-β-carotene	111	0.79	0.50	2.20 (0.82–5.89)	1.08 (0.98–1.18)	0.12
Ln-total β-carotene	221	0.49	0.25	1.64 (1.00–2.66)	1.05 (1.00–1.10)	0.05

^1^ Model 1 is adjusted for CGA, maternal age, and smoking status. ^2^ Model 2 is adjusted for CGA, maternal age, smoking status, and income-to-poverty ratio. ^3^ Odds ratio for abnormal NHS results assuming 1-unit increase in ln(plasma β-carotene). ^4^ Odds ratio for abnormal NHS results assuming 10% increase in plasma β-carotene.

**Table 7:** α-carotene and β-cryptoxanthin intake, maternal plasma, and cord plasma by NHS result.

	Normal NHS Results		Abnormal NHS Results		
	*n*	Median (IQR)	*n*	Median (IQR)	*p*-Value ^1^
α-Carotene					
α-carotene intake (μg/day)	433	477.1 (229.0–812.0)	39	549.6 (221.8–1034.6)	0.61
Maternal α-carotene (μg/L)	345	35.8 (16.9–72.9)	29	32.3 (20.3–99.1)	0.42
Cord α-carotene (μg/L)	280	4.0 (2.3–7.0)	18	5.0 (3.1–17.4)	0.08
β-Cryptoxanthin					
β-cryptoxanthin intake (μg/day)	433	108.3 (59.6–210.1)	39	126.8 (67.8- 232.1)	0.31
Maternal β-cryptoxanthin (μg/L)	346	102.1 (67.6–149.7)	29	128.5 (63.0–239.8)	0.09
Cord β-cryptoxanthin (μg/L)	325	11.6 (7.6–18.6)	24	17.2 (7.3–31.3)	0.09

^1^ Mann–Whitney U test comparing β-carotene nutritional status between normal and abnormal NHS.

## Data Availability

Data are available upon request to the corresponding author. This data is not publicly available due to ethical considerations regarding participant confidentiality.
